# Clinical Spectrum, Etiology, and Complications of Liver Cirrhosis at a Tertiary Care Center in Western India: An Observational Clinic-Based Cohort Study

**DOI:** 10.7759/cureus.97022

**Published:** 2025-11-16

**Authors:** Shabir Badi, Parth Jani, Kuldeepkumar D Viramgama, Dipak Dangar, Krunalkumar B Sarvaiya, Geeta Kanjariya

**Affiliations:** 1 Internal Medicine, All India Institute of Medical Sciences, Rajkot, Rajkot, IND; 2 Internal Medicine, Gujarat Medical Education and Research Society (GMERS) Medical College and Hospital, Sola, Ahmedabad, IND

**Keywords:** alcohol-related liver disease, complications, decompensated cirrhosis, etiology, liver cirrhosis, tertiary care center (india)

## Abstract

Background: Liver cirrhosis, a major global health burden, often presents at a decompensated stage. This study aimed to describe the clinical profile, etiology, and complications of liver cirrhosis at a tertiary care center in Western India, with a focus on identifying treatable causes.

Methods: This observational cohort study was conducted at the Department of Medicine, Gujarat Medical Education and Research Society (GMERS) Medical College and Hospital, Sola, Ahmedabad, between June 1, 2022, and May 31, 2024. One hundred consecutive patients with cirrhosis were enrolled. The study design included an initial cross-sectional analysis at patient presentation and a subsequent one-year prospective follow-up to monitor complications. Data on demographics, clinical presentation, etiology, laboratory parameters, and complications were collected and analyzed using descriptive statistics. The study has been reported in accordance with the Strengthening the Reporting of Observational Studies in Epidemiology* *(STROBE) guidelines.

Results: The mean age was 52.4±14.3 years, with a male predominance (71%; n=71). The most common etiologies were alcohol (66%; n=66), hepatitis B virus (12%; n=12), and non-alcoholic steatohepatitis (11%; n=11). The majority of patients (65%; n=65) had Child-Pugh Class C disease. Common presenting features included abdominal swelling (68%; n=68), jaundice (65%; n=65), and ascites (68%; n=68). Prevalent laboratory abnormalities included anemia (67%; n=67), thrombocytopenia (61%; n=61), hypoalbuminemia (76%; n=76), and an elevated international normalized ratio (INR) (65%; n=65). During follow-up, recurrent ascites (44.6%; n=41 of 92 episodes) was the most common complication leading to readmission. A significant association was found between Child-Pugh Class C and the development of acute kidney injury during follow-up (p<0.01).

Conclusion: This study, based on a tertiary care cohort, confirms alcohol as the leading cause of cirrhosis among hospitalized patients but also highlights a significant burden of viral hepatitis. The high proportion of patients presenting with decompensated disease underscores the critical need for enhanced community-level screening and early intervention strategies.

## Introduction

Chronic liver disease represents a formidable and escalating challenge to global public health, transcending boundaries of age, gender, and geography to impose a substantial burden on healthcare systems worldwide. Cirrhosis, the irreversible end-stage sequel of progressive liver injury, is defined by the pathological triad of hepatic fibrosis, widespread architectural distortion, and the formation of regenerative nodules. This progressive scarring process culminates in a complex and life-threatening clinical syndrome characterized by portal hypertension and progressive hepatic dysfunction, manifesting through a wide spectrum of complications that significantly impair quality of life and survival [1]. The World Health Organization underscores that chronic diseases like cirrhosis are leading contributors to global morbidity and mortality, with their impact steadily rising in both developed and developing nations [1].

The escalating prevalence of cirrhosis is principally fueled by the growing burden of its primary etiological risk factors. These include chronic infections with the hepatitis B virus (HBV) and hepatitis C virus (HCV), as well as sustained and harmful alcohol consumption, which together account for the vast majority of cirrhosis cases globally [2,3]. The clinical course of cirrhosis is often insidious, remaining compensated and asymptomatic for years until a pivotal transition to decompensation occurs. This decompensated phase is marked by the development of severe complications, which define the natural history and prognosis of the disease. A critical and feared complication arising from progressive portal hypertension is variceal hemorrhage, which occurs in 25-40% of cirrhotic patients and is associated with a daunting mortality rate of up to 30% per episode, starkly highlighting the grave prognosis once decompensation sets in [4-7].

Extensive epidemiological research has been conducted to document the trends, etiological spectrum, and clinical complications of liver cirrhosis. A consistent narrative from previous studies, particularly in the Indian context, identifies alcohol as the predominant cause, especially among male populations, closely followed by viral hepatitis B and C [8-11]. These investigations have effectively outlined the common clinical presentation, which typically includes ascites, jaundice, gastrointestinal bleeding, and hepatic encephalopathy, alongside characteristic laboratory abnormalities such as anemia, thrombocytopenia, and hypoalbuminemia [10,12-14]. The literature further solidly establishes the dire complications and poor prognosis associated with advanced disease, particularly in patients classified with high Child-Pugh Scores (CPS), a standardized system for assessing the severity of liver disease [9,15]. The severity of liver cirrhosis was classified using the CPS, which assesses five clinical measures: bilirubin, albumin, international normalized ratio (INR), ascites, and hepatic encephalopathy. Hepatic encephalopathy itself was graded clinically using the West Haven Criteria (WHC) [16].

However, a discernible shortcoming in the existing body of literature is a predominant focus on describing the advanced, decompensated state of the disease and its attendant complications. While characterizing this terminal phase is undeniably crucial for managing end-stage patients, a relative gap exists in emphasizing the paramount importance of identifying and addressing treatable causes at an earlier, potentially compensable stage. The pivotal role of initiating early, targeted treatment, such as lifelong antiviral therapy for HBV, curative direct-acting antivirals for HCV, and complete abstinence in alcohol-related liver disease, in arresting disease progression, preventing complications, and improving long-term survival outcomes requires further emphasis and focused investigation within specific regional patient cohorts.

Therefore, the primary aim of this study is to bridge this gap by conducting a comprehensive, clinic-based profiling of patients with liver cirrhosis. The central objective is to facilitate the identification of treatable etiologies, thereby creating opportunities for intervention before the onset of irreversible decompensation. Our specific objectives are to meticulously document the demographic patterns, clinical presentation, and laboratory parameters of these patients, to delineate the etiological spectrum of cirrhosis in our region, and to identify the various complications they develop during the course of their illness. The scope of this research is a detailed clinical profile analysis of consecutively enrolled patients with cirrhosis at a tertiary care center in Western India, with the ultimate goal of informing strategies for earlier detection and secondary prevention.

## Materials and methods

Study design and reporting

This observational cohort study was conducted at the Department of Medicine, Gujarat Medical Education and Research Society (GMERS) Medical College and Hospital, Sola, Ahmedabad, from June 1, 2022, to May 31, 2024. The design incorporated a baseline cross-sectional analysis of patient profiles, followed by a prospective one-year follow-up to monitor complications. In accordance with the Strengthening the Reporting of Observational Studies in Epidemiology (STROBE) guidelines, and as a descriptive study, a formal a priori power calculation was not performed. Instead, a feasible and representative cohort was established by consecutively enrolling 100 eligible patients over the two-year period. This sample size provides reasonable precision for key estimates; for instance, the observed prevalence of alcohol-related cirrhosis (66%) has a 95% confidence interval of 56-75%.

Ethical considerations

Before starting, the study received full approval from the Institutional Ethics Committee of GMERS Medical College and Civil Hospital, Sola (approval number: GMERSMCS/IEC/25/2022). Every participant provided written informed consent before joining the study, and the research adhered to the ethical standards outlined in the Declaration of Helsinki.

Participants

The study group included 100 patients diagnosed with liver cirrhosis who were admitted to the medicine ward. Participants were recruited consecutively as they were admitted, provided they met the specific criteria. To be included, individuals had to be over 18 years of age with a confirmed diagnosis of liver cirrhosis. Exclusion reasons included being under 18, declining to participate, or having a chronic liver condition not related to cirrhosis, such as congenital hepatic fibrosis.

Diagnostic criteria

Diagnosis of Cirrhosis

The diagnosis of liver cirrhosis was established using a composite of clinical, laboratory, and radiological criteria, a pragmatic approach endorsed for settings where transient elastography is unavailable. The diagnosis of cirrhosis required the presence of all three of the following components: (1) at least one clinical sign of chronic liver disease (such as jaundice or palmar erythema), (2) at least one sign of portal hypertension (such as ascites or splenomegaly), and (3) an abdominal ultrasound demonstrating at least two suggestive features (including a nodular liver surface, coarse parenchymal echotexture, caudate lobe hypertrophy, or portosystemic collateral vessels). A liver biopsy was not mandatory for enrollment and was performed only in cases where the diagnosis remained uncertain [17,18].

Ascertainment of Etiology

Etiology was determined using the following predefined, standardized criteria: First is alcohol-related liver disease, meaning significant alcohol intake (>80 g/day for men or >40 g/day for women for ≥10 years), after excluding other causes. If a precise quantitative history was unavailable, the diagnosis was made by a senior gastroenterologist based on a consistent history of hazardous drinking. Second is HBV, meaning a positive hepatitis B surface antigen (HBsAg) test. Third is HCV, meaning a positive anti-HCV antibody test confirmed by detectable HCV RNA. Fourth is non-alcoholic steatohepatitis (NASH), meaning a diagnosis of exclusion in patients with at least two metabolic risk factors (e.g., type 2 diabetes, hypertension, BMI >25 kg/m²), in the absence of significant alcohol consumption (<20 g/day) and other known liver diseases. Fifth is autoimmune hepatitis, meaning positive serological markers (antinuclear antibody (ANA), anti-smooth muscle antibody (ASMA)) with a compatible clinical presentation. Sixth is other etiologies: Wilson's disease and others were investigated on a case-by-case basis using appropriate tests. Cryptogenic cirrhosis was diagnosed only after all common causes were systematically ruled out [17,18].

Data collection and follow-up procedures

Upon enrollment, a thorough baseline assessment was performed. Data on demographics, clinical presentation, etiology, laboratory parameters, and imaging findings were collected from detailed patient histories, physical examinations, and standard tests performed in the hospital's central laboratory.

Patients were subsequently followed for one year to track predefined cirrhosis-related complications. The follow-up was conducted through passive surveillance of hospital readmission records at our tertiary care center. As the primary referral center for the region, it is standard practice for patients with significant complications to return to our facility for management. Active follow-up via telephone or scheduled outpatient visits was not performed.

Complication outcomes were defined as follows: (1) recurrent ascites, readmission requiring therapeutic paracentesis or intensification of diuretic therapy for diuretic-resistant or diuretic-intractable ascites, (2) hepatic encephalopathy, readmission with an episode of mental status alteration graded ≥II on the WHC, (3) acute kidney injury (AKI), readmission with a diagnosis of AKI as per the International Club of Ascites (ICA) criteria (increase in serum creatinine by ≥0.3 mg/dL within 48 hours or a ≥50% increase from baseline), (4) variceal bleeding, readmission with hematemesis or melena with endoscopic confirmation of bleeding from esophageal or gastric varices, (5) spontaneous bacterial peritonitis (SBP), diagnostic paracentesis confirming an ascitic fluid polymorphonuclear (PMN) cell count >250 cells/mm³, and (6) hepatorenal syndrome (HRS), development of HRS as defined by international criteria (ICA-AKI) in a patient with advanced liver disease [18].

For the analysis, all distinct readmission events meeting the above criteria were counted as separate episodes. Therefore, the complication rates represent the total burden of hospitalizations for these indications within the cohort, rather than the proportion of patients who experienced at least one event.

Statistical analysis

For statistical analysis, the team used IBM SPSS Statistics for Windows, V. 28.0 (IBM Corp., Armonk, NY, USA). They summarized categorical data as frequencies (n) and percentages (%) and continuous data as means with standard deviations. Since the analysis included 100 patients, frequency (n) and percentage (%) values are reported for all categorical variables in the main text and media items. To test for associations between categorical variables, the chi-squared test was applied, with Fisher's exact test used where suitable. A p-value of <0.05 was considered statistically significant.

## Results

Participant flow

During the study period, 125 patients were assessed for eligibility. Fifteen were excluded (five were under 18 years, seven had chronic liver disease not due to cirrhosis, and three declined to participate). A total of 110 patients were enrolled and constituted the baseline cross-sectional cohort for the analysis of initial presentation, etiology, and disease severity. Of these, 10 patients were lost to follow-up after discharge. Therefore, 100 patients completed the one-year follow-up and were included in the prospective analysis of complications. Of these, 92 complication events were recorded in 78 patients during the one-year follow-up period.

Demographic characteristics

The demographic profile of the study participants is summarized in Table 1. The mean age of the cohort was 52.4±14.3 years. The majority of patients (39%; n=39) were aged over 60 years, followed by those in the 41-50-year age group (24%; n=24). The study population was predominantly male (71%; n=71), resulting in a male-to-female ratio of 2.4:1.

**Table 1:** Age and gender distribution of the study participants (n=100)

Characteristic	Category	Number of patients	Percentage
Age group (years)	21-30	4	4%
	31-40	22	22%
	41-50	24	24%
	51-60	11	11%
	>60	39	39%
Gender	Male	71	71%
	Female	29	29%

Clinical presentation

The most common presenting symptoms and signs are detailed in Table 2. Abdominal swelling (68%; n=68), pedal edema (65%; n=65), and yellowish discoloration of eyes (65%; n=65) were the most frequent symptoms. On clinical examination, the most prevalent signs were ascites (68%; n=68), jaundice (65%; n=65), pallor (63%; n=63), and asterixis (56%; n=56).

**Table 2:** Clinical symptoms and signs at presentation (n=100)

	Number of patients	Percentage
Symptoms		
Abdominal swelling	68	68%
Pedal edema	65	65%
Yellowish discoloration of eyes	65	65%
Constipation	44	44%
Disorientation	30	30%
Signs		
Ascites	68	68%
Jaundice	65	65%
Pallor	63	63%
Asterixis	56	56%
Splenomegaly	47	47%

Etiology

The etiological distribution of liver cirrhosis is presented in Table 3. Alcohol was the leading cause, accounting for 66% (n=66) of cases. HBV infection was the second most common cause (12%; n=12), followed by NASH (11%; n=11) and HCV infection (6%; n=6).

**Table 3:** Etiology of liver cirrhosis (n=100) HBV: hepatitis B virus; NASH: non-alcoholic steatohepatitis; HCV: hepatitis C virus

Etiology	Number of patients	Percentage
Alcohol	66	66%
HBV	12	12%
NASH	11	11%
HCV	6	6%
Wilson's disease	1	1%
Other	4	4%

Laboratory parameters

Hematological and biochemical abnormalities are summarized in Table 4. Anemia (hemoglobin <12 g/dL) was present in 67% (n=67) of patients, and thrombocytopenia (platelet count <1.5 lakh/μL) was observed in 61% (n=61). Liver function tests were markedly abnormal, with elevated serum glutamic-oxaloacetic transaminase (SGOT) and serum glutamic-pyruvic transaminase (SGPT) levels in 84% (n=84) and 75% (n=75) of patients, respectively. Hyperbilirubinemia (total bilirubin >1.2 mg/dL) was found in 78% (n=78) of the cohort. Hypoalbuminemia (serum albumin <3.5 g/dL) was present in 76% (n=76) of patients, with 65% (n=65) having a severe deficiency (<2.5 g/dL). Coagulopathy was common, with 65% (n=65) of patients having an INR >2.3.

**Table 4:** Laboratory abnormalities in the study participants *Hypoalbuminemia with 65% having a severe deficiency (<2.5 g/dL) SGOT: serum glutamic-oxaloacetic transaminase (aspartate aminotransferase (AST)); SGPT: serum glutamic-pyruvic transaminase (alanine aminotransferase (ALT)); INR: international normalized ratio

Parameter (normal range)	Abnormality	Number of patients	Percentage
Hemogram			
Hemoglobin (12-16 g/dL)	<12 g/dL	67	67%
Platelets (1.5-4.5 lakh/μL)	<1.5 lakh/μL	61	61%
Liver function tests			
SGOT (5-40 U/L)	>40 U/L	84	84%
Total bilirubin (0.2-1.2 mg/dL)	>1.2 mg/dL	78	78%
SGPT (5-40 U/L)	>40 U/L	75	75%
Serum albumin (3.5-5.2 g/dL)	<3.5 g/dL	76	76%
	<2.5 g/dL*	65*	65%
Coagulation profile			
INR (<1.2)	>2.3	65	65%

Disease severity and complications

The severity of liver disease, as classified by the CPS, is shown in Table 5. The majority of patients (65%; n=65) had Class C disease, indicating decompensated cirrhosis, while 11% (n=11) had Class B and 24% (n=24) had Class A.

**Table 5:** Disease severity by Child-Pugh Classification (n=100)

Child-Pugh Class	Number of patients	Percentage
A (compensated)	24	24%
B (decompensated)	11	11%
C (decompensated)	65	65%

Hepatic encephalopathy was present in 76% (n=76) of patients at the time of enrollment. According to the WHC, 32% (n=32) were in Grade II and 30% (n=30) were in Grade III (Table 6).

**Table 6:** Grading of hepatic encephalopathy (West Haven Criteria) (n=100)

West Haven Grade	Number of patients	Percentage
0	24	24%
I	11	11%
II	32	32%
III	30	30%
IV	3	3%

All patients (100%; n=100) had ultrasonographic evidence of a shrunken liver. Ascites was detected in 68% (n=68) of patients, and splenomegaly was present in 47% (n=47). During a one-year follow-up period, the most common complication leading to hospital readmission was recurrent ascites (44.56%; n=41 of 92 episodes), followed by others like hepatic encephalopathy (22.83%; n=21) and AKI (21.74%; n=20) (Table 7). During the prospective one-year follow-up period, 92 complication events were recorded in 78 patients.

**Table 7:** Complications during one-year follow-up (n=92 episodes)

Complication	Frequency	Percentage
Recurrent ascites	41	44.56%
Hepatic encephalopathy	21	22.83%
Acute kidney injury	20	21.74%
Hematemesis	5	5.44%
Spontaneous bacterial peritonitis	3	3.26%
Hepatorenal syndrome	2	2.17%

Association analysis

A chi-squared test revealed a significant association between the etiology of cirrhosis and the presence of hepatic encephalopathy at admission (p<0.05). Patients with alcohol-related cirrhosis had a higher prevalence of hepatic encephalopathy (80.3%; n=53) compared to those with non-alcoholic etiologies (67.6%; n=23). Furthermore, Fisher's exact test indicated a significant association between Child-Pugh Class C and the development of AKI during follow-up (p<0.01). 

## Discussion

This clinic-based cohort study found a comprehensive profile of the clinical, etiological, and laboratory characteristics of 100 patients with liver cirrhosis at a tertiary care center in Western India. The key findings indicate a predominance of male patients and an older age group, with alcohol consumption being the leading etiology. A significant majority of patients presented with advanced disease, as evidenced by a high prevalence of complications like ascites and hepatic encephalopathy, and were classified as Child-Pugh Class C.

The demographic profile of our cohort, with a male predominance (71%; n=71) and a high median age, aligns consistently with numerous national studies [10-12]. This recurring pattern is largely attributable to higher rates of alcohol consumption and exposure to other risk factors among males in the region. The most common presenting complaints and clinical signs, abdominal distension (ascites), jaundice, and splenomegaly, are classic manifestations of decompensated cirrhosis and portal hypertension, findings that are remarkably consistent with those reported by other Indian studies [10-12].

A critical finding of our study was the etiological distribution, with alcohol accounting for 66% (n=66) of cases. While this confirms alcohol as the primary driver of cirrhosis in our population, as seen in other Indian studies [10,11], we observed a notably higher prevalence of hepatitis B (12%; n=12) and hepatitis C (6%; n=6) compared to some reports [10]. This discrepancy is not a contradiction but rather a unique characteristic of our cohort, likely explained by our institution's role as a Model Treatment Centre (MTC) for viral hepatitis, which attracts a higher number of referred patients with HBV and HCV.

The laboratory abnormalities further paint a picture of advanced hepatocellular dysfunction. The high prevalence of anemia (67%; n=67), thrombocytopenia (61%; n=61), hypoalbuminemia (76%; n=76), elevated INR (65%; n=65), and hyperbilirubinemia (78%; n=78) closely mirrors the findings of other studies [13,14]. These results mechanistically reflect the loss of the liver's synthetic function, portal hypertensive sequelae like splenic sequestration, and the general catabolic state of chronic disease.

This extensive burden of disease is succinctly captured by the CPS, where 65% (n=65) of our patients were in Class C. This finding indicates that our study population represents a cohort with end-stage liver disease, accounting for the high rate of complications observed during follow-up, with ascites being the most common. Our statistical analysis further strengthens this, revealing a significant association between Child-Pugh Class C and the development of AKI, a known and serious complication in decompensated cirrhosis [14]. This finding is strongly supported by a contemporaneous regional study by Parmar et al., which identified renal dysfunction as a prevalent complication in cirrhotic patients, with upper gastrointestinal hemorrhage and infections being the primary precipitants [19].

New findings and limitations of the study

A key insight from this study is the demonstration of how a hospital's specialized role (as an MTC) can significantly influence the observed etiological pattern of a disease within its patient population. Furthermore, the statistical confirmation of the link between advanced Child-Pugh Class and renal complications adds a layer of analytical depth to the descriptive profile.

However, these strengths must be balanced against several limitations. First, the single-center design and consecutive sampling method limit the generalizability of our findings to the broader community. Second, the sample size, though adequate for a descriptive study, may have been underpowered for more complex multivariate analysis. Third, as an observational study, it is susceptible to unmeasured confounding factors. Finally, despite a one-year follow-up, 10 patients were lost, potentially introducing attrition bias.

Recommendations

Based on our findings, we propose the following recommendations: First is enhanced screening and referral. The high prevalence of advanced disease at presentation calls for strengthened community-level screening programs for alcohol use disorder and viral hepatitis. Second is protocol-based management. The high frequency of complications warrants the implementation of standardized, protocol-driven management pathways. Third is focus on renal function. Given the significant association with AKI, vigilant monitoring of renal function is crucial. Fourth is future research. Multi-center prospective studies with larger sample sizes are needed to validate our etiological findings and investigate long-term outcomes.

## Conclusions

The findings of this study delineate a clinical profile characterized by the late presentation of liver cirrhosis among a tertiary care cohort, with a significant majority of patients already exhibiting decompensated disease upon initial contact. The etiological landscape is dominated by alcohol-related liver disease, yet a considerable burden of cirrhosis attributable to viral hepatitis is evident, a distribution potentially influenced by the institution's specific role as a referral center. The high prevalence of Child-Pugh Class C disease (65%), alongside frequent complications such as ascites, hepatic encephalopathy, and a significant association between advanced disease and AKI, paints a stark picture of the advanced stage at which patients enter the tertiary care system.

This pattern of advanced disease at diagnosis underscores a critical disconnect in the healthcare pathway, where opportunities for early intervention within the community are being missed. Consequently, these observations highlight an imperative for a paradigm shift in public health strategy towards proactive screening and early secondary prevention. Enhancing community-level detection of modifiable risk factors, particularly hazardous alcohol consumption and chronic viral hepatitis infections, is essential to alter the natural history of this disease. Implementing such measures is a fundamental prerequisite for reducing the incidence of decompensated cirrhosis and mitigating its associated morbidity and mortality.
